# Remarkable remission of a tumor‐stage mycosis fungoides on the scalp by single fraction palliative radiotherapy

**DOI:** 10.1002/ccr3.6333

**Published:** 2022-09-14

**Authors:** Atsuto Katano

**Affiliations:** ^1^ Department of Radiology University of Tokyo Hospital Tokyo Japan

**Keywords:** mycosis fungoides, palliative therapy, radiotherapy, single fraction

## Abstract

For tumor‐stage mycosis fungoides, systemic therapy in combination with skin‐directed local therapy is the mainstay of treatment. Here, we report a case where a tumor‐stage mycosis fungoides of the scalp showed a remarkable response to single fraction palliative radiotherapy.

## 1 CLINICAL CASE

**FIGURE 1:**
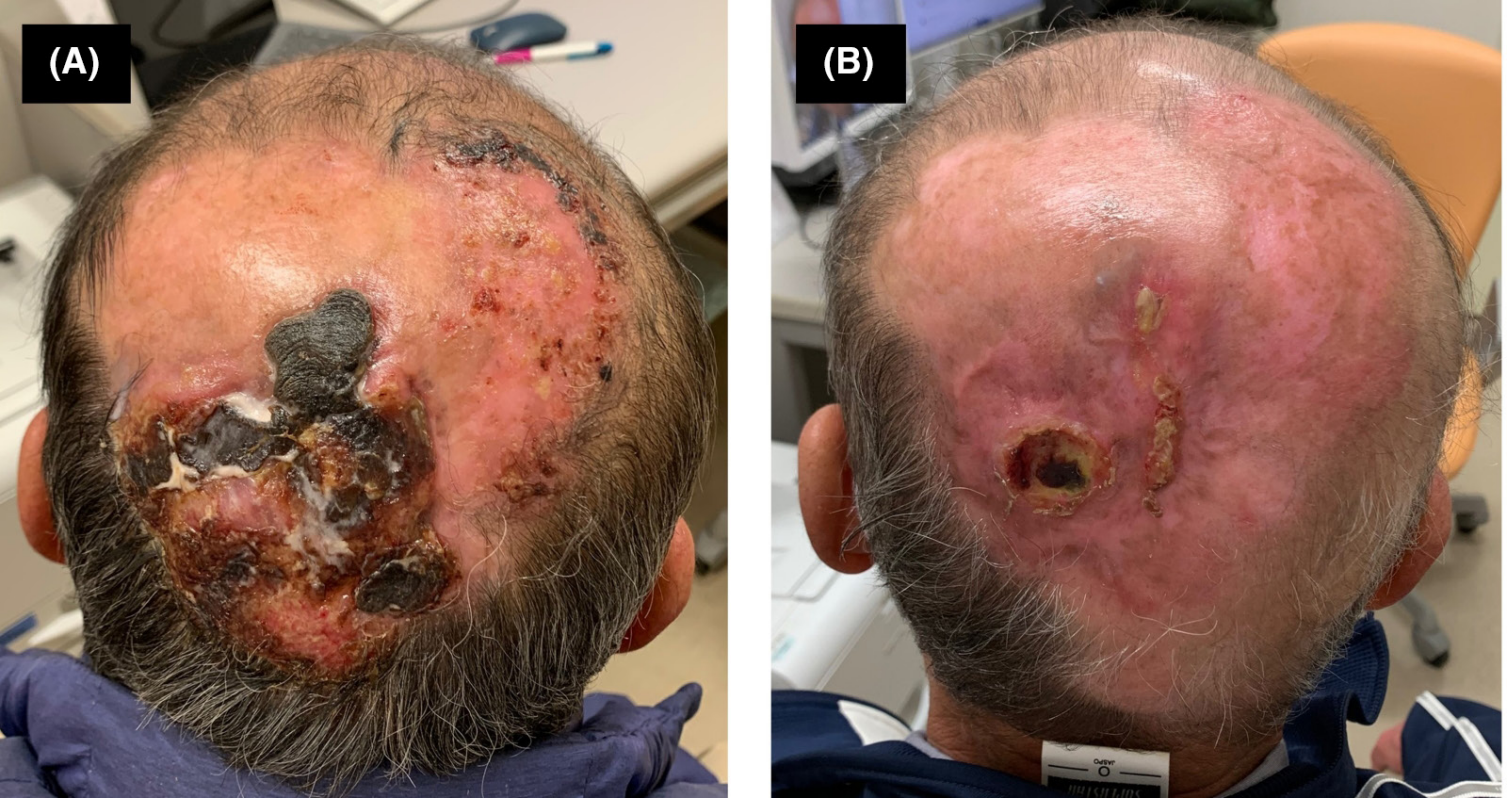
Mycosis fungoides tumor over 10 cm in diameter with black tissue in the scalp. (A) Before radiotherapy. (B) Four months after single fraction palliative radiotherapy.

A man in his 60s with tumor‐stage mycosis fungoides complained of itching and oozing in his scalp during retinoid therapy consisting of a daily dose of 40 mg etretinate without any monoclonal antibody therapies. He had been diagnosed with mycosis fungoides 5 years ago and had received several treatments, including narrowband ultraviolet B phototherapy, topical corticosteroid, and oral bexarotene administration. The tumor was located in the occipital region, being over 10 cm in diameter with black tissue when he was referred to our department (Figure 1A). Skin‐directed electron radiotherapy was performed as a palliative treatment for the occipital tumor. The prescribed dose was 8 Gy in a single fraction, and an electron energy of 6 MeV was chosen with a bolus thickness of 5 mm onto the tumor. After 4 months, the tumor significantly regressed without any adverse events related to radiotherapy (Figure 1B).

Neelis et al.^1^ also reported good efficacy of low‐dose palliative radiotherapy for mycosis fungoides. However, the median size of the lesion that they treated was significantly smaller (4 cm) than the one in our case. Our report extends this observation by demonstrating that single fraction palliative radiation can be effective even in the case of a larger tumor‐stage mycosis fungoides.

## AUTHOR CONTRIBUTION

The author confirms solely contributions and responsibilities for all aspect of this manuscript.

## FUNDING INFORMATION

None declared.

## CONFLICT OF INTEREST

None declared.

## CONSENT

Written informed consent was obtained from the patient to publish this report in accordance with the journal's patient consent policy.

## Data Availability

No data are available.
